# A giant testicular tumor requiring skin valvuloplasty

**DOI:** 10.1007/s13691-026-00846-6

**Published:** 2026-04-07

**Authors:** Shigeaki Nakazawa, Shunki Nakagawa, Yu Ishizuya, Yoshiyuki Yamamoto, Kentaro Takezawa, Taigo Kato, Koji Hatano, Yoichi Kakuta, Atsunari Kawashima, Norio Nonomura

**Affiliations:** Department of Urology, Graduate School of Medicine, The University of Osaka, 2-2 Yamadaoka, Suita, Osaka 565-0871 Japan

**Keywords:** Giant testicular tumor, Nonseminoma, Skin valvuloplasty

## Abstract

Giant testicular tumors, typically defined as those with volumes exceeding ten times the normal testicular volume, are extremely rare in developed countries. However, a relatively high number of such cases have been reported in Japan. Social stigma and lack of awareness often contribute to delayed medical consultation, resulting in advanced disease requiring complex surgical interventions. Despite its aggressive presentation, testicular cancer is curable when diagnosed early, highlighting the importance of public education and awareness. A 45-year-old man presented with hemorrhagic shock due to bleeding from massively enlarged left scrotum. He first noticed painless scrotal swelling 3 years earlier, which rapidly progressed over the previous 6 months. Imaging revealed a 34-cm heterogeneous testicular mass with bilateral inguinal lymphadenopathy. Tumor marker levels were significantly elevated (alpha-fetoprotein: 689 ng/mL, lactate dehydrogenase: 6,566 IU/L). Emergency radical orchiectomy and bilateral inguinal lymph node dissection were performed. Due to extensive skin necrosis, vacuum-assisted closure therapy was initiated, followed by delayed skin valvuloplasty. Histopathological analysis confirmed nonseminomatous germ cell tumor (Stage IIIA), and adjuvant vincristine, ifosfamide, and cisplatin chemotherapy was administered. The patient remains recurrence-free 2 years postoperatively. Although rare, giant testicular tumors require prompt diagnosis and multidisciplinary management. Early detection is essential for preventing life-threatening complications and enabling curative treatment. Public awareness of testicular self-examination and high curability of testicular cancer can significantly reduce delays in seeking medical attention.

## Introduction

Although rare across the lifespan, testicular cancer is the most frequently diagnosed malignancy in men aged 15–35 years [1]. Most patients become aware of a painless scrotal swelling and promptly seek medical attention. However, in some cases, lack of disease awareness and hesitation to visit medical institutions can lead to prolonged neglect of the condition. In the current era of advanced healthcare, the presentation of large testicular tumors is exceedingly uncommon.

The primary treatment for testicular tumors is inguinal radical orchiectomy. However, in cases of massive tumors, an extended surgical approach, including reconstructive procedures, may be required. Herein, we report a case of a giant testicular tumor weighing > 5 kg that required skin valvuloplasty along with a review of the relevant literature.

## Case report

**Fig. 1:**
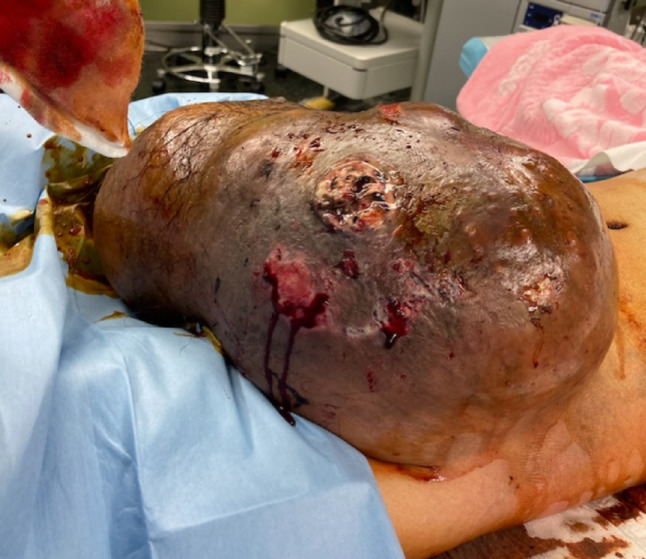
At admission, the scrotum is enlarged with the self-destructive skin area and shows continuous bleeding

A 45-year-old man was referred to the emergency department with active bleeding from a grossly enlarged left scrotum. He experienced hemorrhagic shock upon arrival and was transferred to our facility while receiving a transfusion. Physical examination revealed massive scrotal distension and ulcerated erythematous skin. The penis was buried within the mass, and bleeding from the skin ulceration persisted (Fig. 1). This was his first medical evaluation. He first noticed painless left scrotal swelling 3 years earlier, which had accelerated over the preceding 6 months. His medical and surgical histories were unremarkable.

**Fig. 2:**
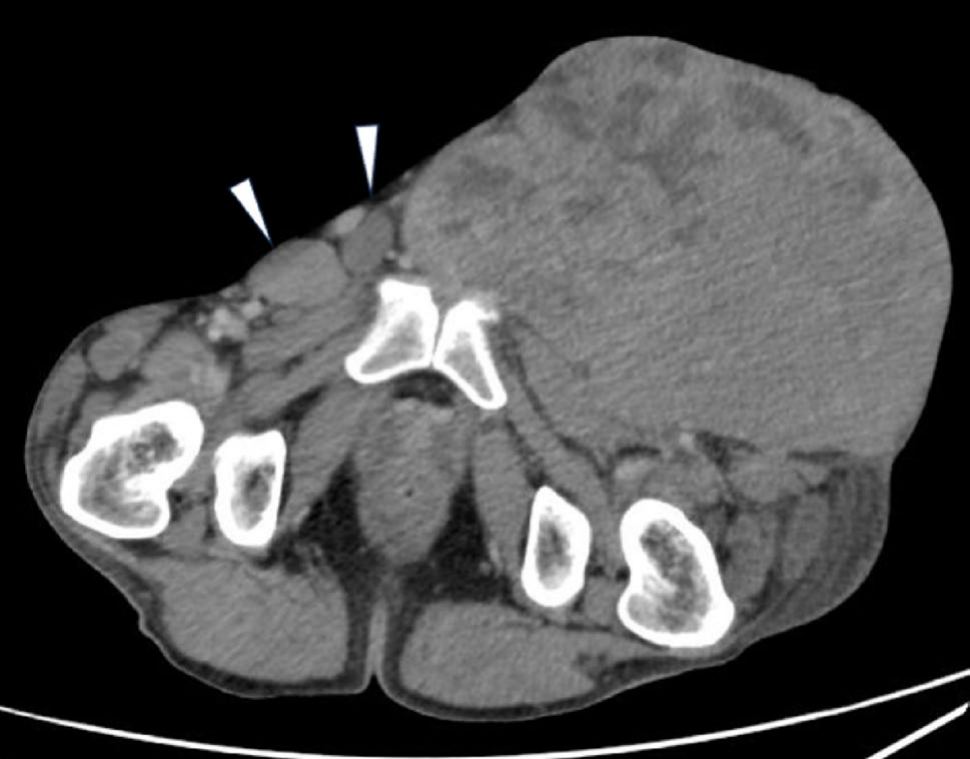
Contrast-enhanced computed tomography shows a heterogenous left testicular tumor measuring 34 × 23 × 16 cm and enlarged bilateral inguinal lymph nodes (arrows, left: 3 × 2 cm, right: 4 × 3 cm)

Laboratory findings demonstrated elevated levels of the following tumor markers: human chorionic gonadotropin (HCG) 15.4 mIU/mL, alpha-fetoprotein (AFP) 689 ng/mL, and lactate dehydrogenase (LDH) 6,566 IU/L. Contrast-enhanced computed tomography revealed a heterogeneous left testicular mass measuring 34 × 23 × 16 cm along with bilateral inguinal lymphadenopathy (left: 3 × 2 cm, right: 4 × 3 cm) (Fig. 2). Distant metastases were not observed.

**Fig. 3:**
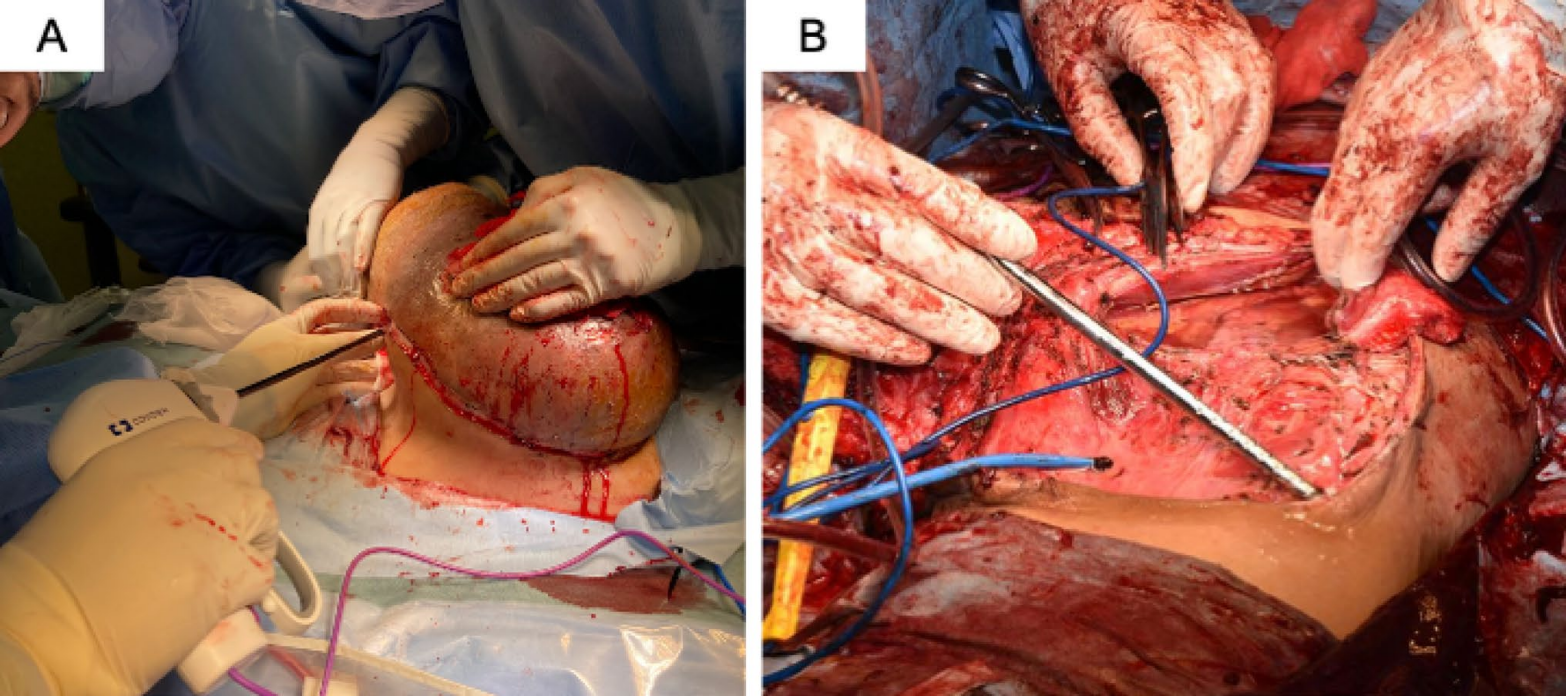
Intraoperative findings. A circumferential skin incision is made around the tumor (**A**). The skin defects after tumor removal (**B**)

**Fig. 4:**
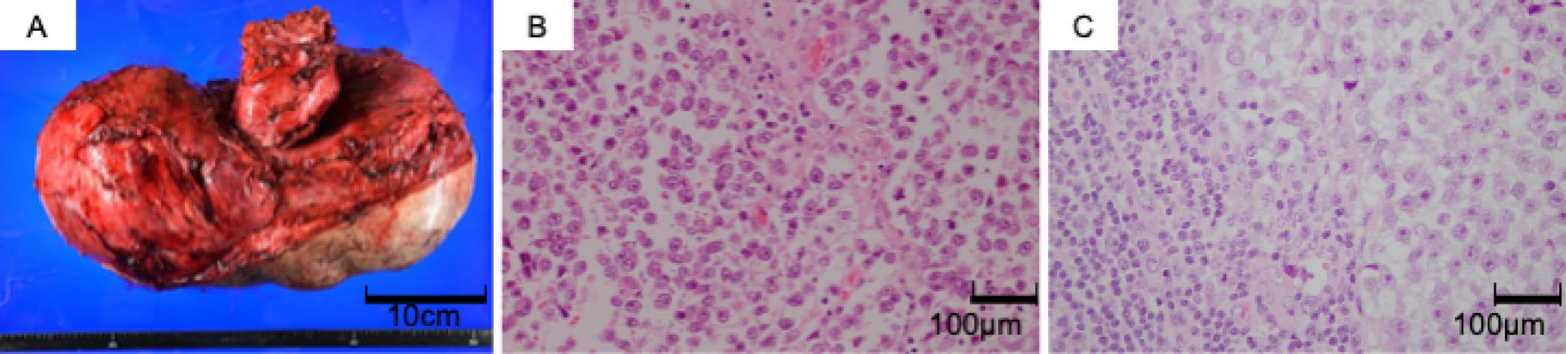
Pathological findings. Resected specimen (**A**). Hematoxylin and eosin staining of testicular tumor (**B)** and inguinal lymph node tissues (**C**). For both tissues, the pathological diagnosis made was of seminoma

Emergency surgery was performed on the following day. En bloc excision of the tumor, including the necrotic scrotal skin, was performed. Anticipating extensive skin defects, secondary skin valvuloplasty was planned. A circumferential incision was made (Fig. 3 A) and the posterior tumor surface was dissected from the puborectalis and adductor muscles. The inguinal ligament was transected, and left radical orchiectomy was performed without primary closure (Fig. 3B). Bilateral inguinal lymph node dissection was performed. The procedure lasted 336 min, with an estimated blood loss of 2,250 mL. The excised mass measured 38 cm in diameter and weighed 5.690 kg (Fig. 4 A). Histology confirmed a seminoma with bilateral inguinal lymph node metastases (Fig. 4B, 4 C); however, the clinical diagnosis made was of nonseminoma owing to markedly elevated AFP levels, in accordance with the National Comprehensive Cancer Network criteria [2]. Lymphovascular and spermatic cord invasions were noted; however, the margins were clear at the cord stump. The tumor marker levels rapidly normalized postoperatively. The final staging was nonseminoma, pT3N0M1aS0, and Stage IIIA. According to the IGCCCG classification [3], this case was categorized as good-risk nonseminomatous germ cell tumor.

**Fig. 5:**
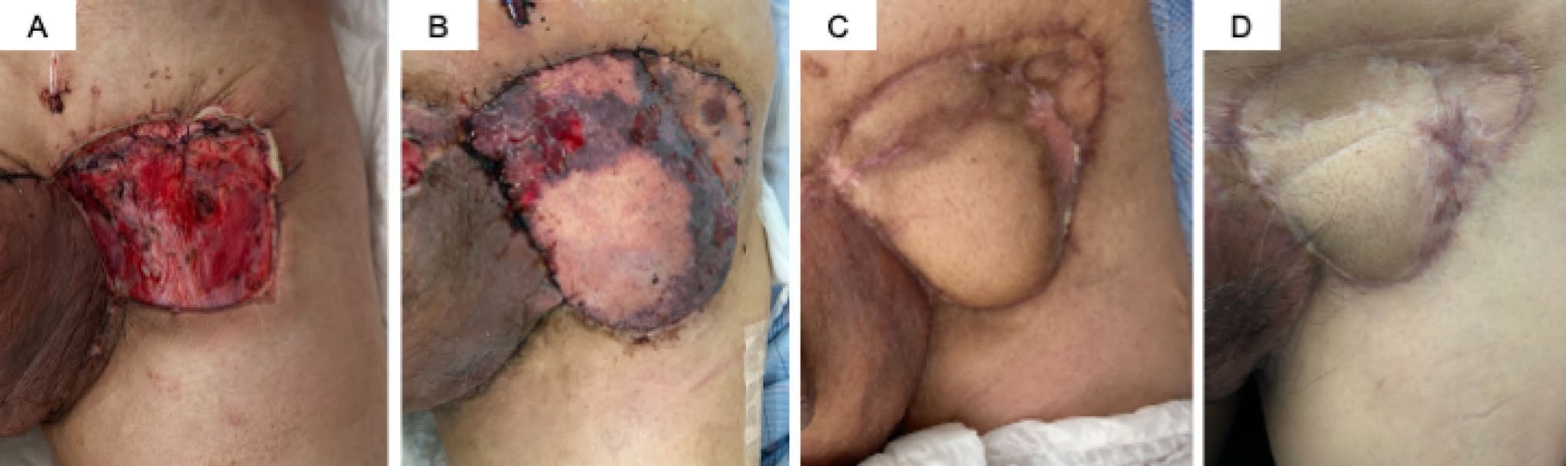
Skin defect before and after valvuloplasty. Images taken (**A**) 28 days, (**B**) 35 days, (**C**) 97 days, (**D**) and 412 days after high orchidectomy

**Fig. 6:**
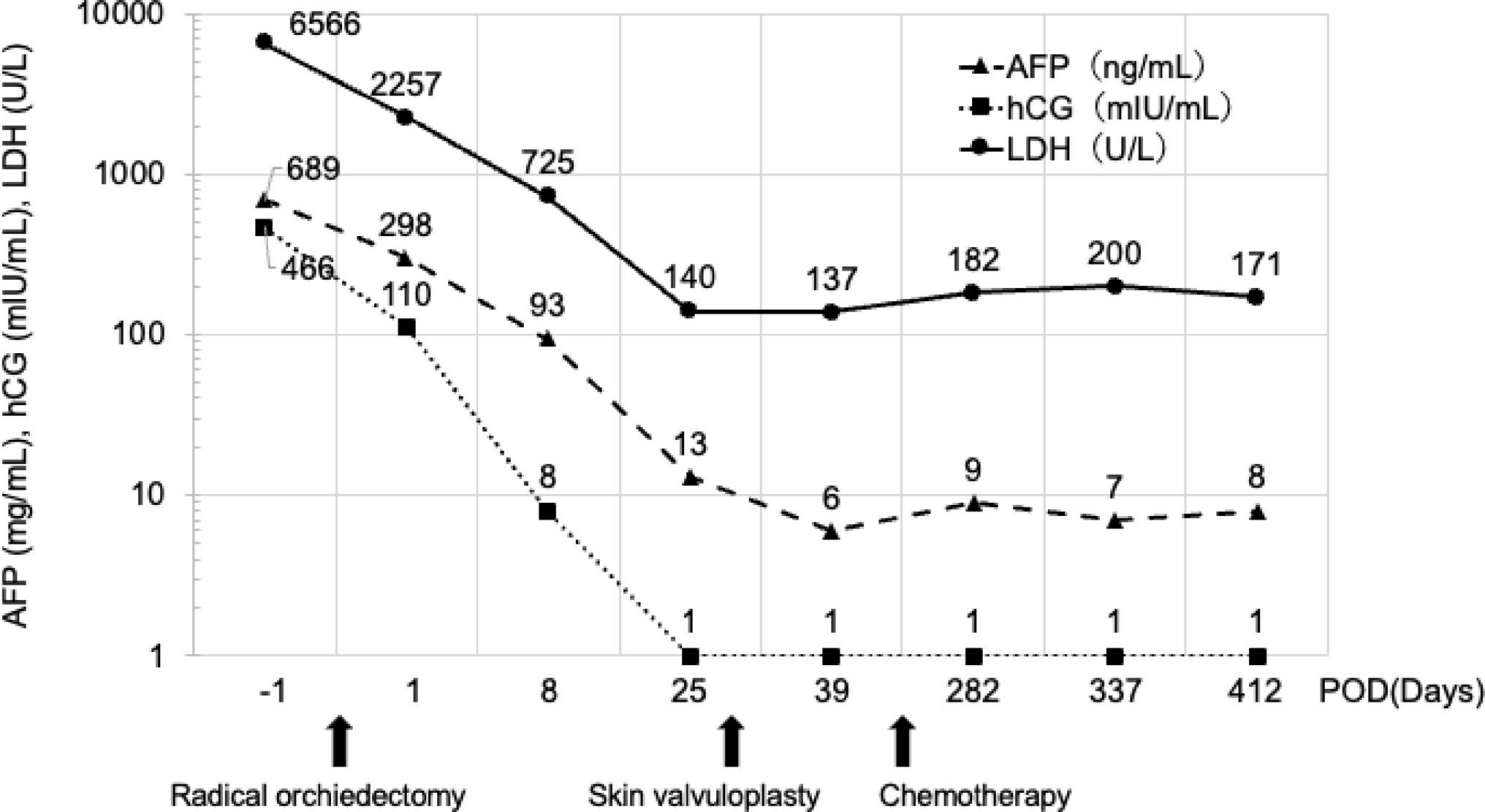
Tumor marker levels before and after high orchiectomy. All tumor marker levels decreased rapidly postoperatively and became negative at 25 days postoperatively.VIP, vincristine, ifosfamide, and cisplatin; POD, postoperative day.

The skin defect measured approximately **8 × 9 cm**, and vacuum-assisted closure (VAC) therapy was initiated on postoperative day 2 to promote granulation and control local contamination. On postoperative day 28, **a plastic surgeon designed and elevated a 7-cm-wide anterolateral thigh (ALT) flap from the left thigh**, which was used for secondary reconstruction of the scrotal skin defect. The postoperative course was uneventful, and satisfactory wound healing was achieved (Figs. 5 A–5D). Adjuvant chemotherapy with two cycles of vincristine, ifosfamide, and cisplatin (VIP) was initiated on postoperative day 40. At the 2-year follow-up, the patient remained recurrence-free (Fig. 6).

## Discussion

Giant testicular tumors, defined as masses with volumes exceeding 10 times the normal testicular volume, are exceptionally rare, with only a few cases reported globally [4–7]. Delayed presentation is frequently associated with stigma and psychological distress. In our case, the patient had been socially withdrawn and delayed seeking medical care for 3 years.

We conducted a literature search using PubMed and Ichushi-Web with the keywords ‘giant testicular tumor,’ ‘giant seminoma,’ and ‘giant germ cell tumor.’ The majority of previously published cases originated from Japan and were primarily individual case reports [8–12]. Potential contributing factors include delayed medical consultation related to cultural hesitation toward genital examination, social isolation, and disparities in healthcare access. Table 1 summarizes the 68 cases of giant testicular tumors reported in Japan. The median patient age was 37 years, and the median specimen weight was 1800 g. Histopathological findings indicated that 71% of the cases were seminomas, and 12% were ultimately categorized as nonseminomas based on elevated AFP levels.

**Table 1 Characteristics of patients in 68 cases of giant testicular tumors reported in Japan:** .

		N=68
Age, years		36.5 (0-81)
Location, left/right		32(47%) / 36(53%)
Weight, g		1,770 (432-7,000)
Pathological diagnosis	Seminoma	48 (71%)
	Nonseminoma	19 (28%)
	Rhabdomyosarcoma	1 (1.5%)
Final diagnosis	Seminoma	39 (57%)
	Nonseminoma	28 (41%)
	Rhabdomyosarcoma	1 (1.5%)
Site of metastasis at the time of initial examination		37(54%)
Lymph node	Retroperitoneal	27 (40%)
	Inguinal	3 (4.4%)
	Cervical	2 (2.9%)
	Mediastinal	4 (5.9%)
Lung		10 (15%)
Bone		4 (5.9%)
Median (range), or n (%)		

Retroperitoneal lymph node involvement was observed in 55% of the cases. Distant metastases commonly involved the lungs (44%), mediastinum (17%), bones (17%), inguinal nodes (13%), and cervical nodes (9%). To the best of our knowledge based on our literature search, we were unable to identify any prior reports of contralateral inguinal lymph node metastasis in giant testicular tumors.

Radical orchiectomy remains both diagnostic and therapeutic and is recommended for initial management unless systemic chemotherapy is urgently needed.　Among the cases in the literature, 45% of patients underwent neoadjuvant chemotherapy, which likely reflects surgical inoperability at presentation. Only a few patients required reconstructive skin procedures, and there is no consensus as to whether immediate or delayed valvuloplasty is superior. We employed VAC therapy to minimize the risk of infection in the pubic area and reduce skin defects before performing delayed reconstruction. Compared with conventional gauze dressing, VAC therapy accelerates granulation, decreases bacterial burden, and reduces wound edema, thereby facilitating more effective preparation for skin grafting or flap reconstruction.

Although this patient had Stage IIIA disease, surgical complete remission (CR) was achieved after complete resection of the primary tumor and metastatic lymph nodes. For Stage IIIA patients who achieve surgical CR, current guidelines do not provide definitive recommendations, and both postoperative surveillance and adjuvant chemotherapy remain acceptable options, as evidence is limited. Therefore, we referred to the rationale derived from high-risk Stage I nonseminoma—not as a staging substitute, but as a conceptual framework—where adjuvant chemotherapy is used after complete resection, to guide management in this rare clinical scenario. Although BEP is the standard regimen, VIP is an accepted alternative when bleomycin is contraindicated. Based on the patient’s age and smoking history, two cycles of VIP were selected to avoid bleomycin-induced pulmonary toxicity.

Giant testicular tumors are uncommon in high-resource countries but continue to be reported in disproportionately high numbers in Japan. Delayed presentation is a well-recognized issue in testicular cancer and is often associated with a lack of awareness or hesitation to seek medical care. Prior studies have shown that testicular self-examination (TSE) and increased awareness of scrotal abnormalities may facilitate earlier presentation and reduce the likelihood of advanced-stage disease [13]. In the context of giant testicular tumors, which frequently arise from prolonged neglect, improved education on scrotal abnormalities and the high curability of testicular cancer may help mitigate delays in diagnosis.

## Conclusions

Giant testicular tumors are uncommon in high-resource countries but continue to be reported disproportionately in Japan. Early recognition, timely surgical management, and appropriate adjuvant therapy are essential for achieving favorable outcomes. Public health initiatives promoting testicular self-examination and awareness of the high curability of testicular cancer are needed to prevent delayed diagnoses.

## Data Availability

The data supporting the findings of this study are available from the corresponding author upon reasonable request.
